# Brimonidine eye drops reveal diminished sympathetic pupillary tone in comatose patients with brain injury

**DOI:** 10.1007/s00701-023-05569-8

**Published:** 2023-04-04

**Authors:** Elisabeth Waldemar Jakobsen, Vardan Nersesjan, Simon Sander Albrechtsen, Marwan H. Othman, Moshgan Amiri, Niels Vendelbo Knudsen, Merlin D. Larson, Christian Hassager, Kirsten Møller, Jesper Kjaergaard, Daniel Kondziella

**Affiliations:** 1 Department of Neurology, Rigshospitalet, Copenhagen University Hospital, Blegdamsvej 9, DK-2100 Copenhagen, Denmark; 2 Department of Neuroanesthesiology, Rigshospitalet, Copenhagen University Hospital, Copenhagen, Denmark; 3 Department of Anesthesiology, University of California San Francisco, San Francisco, CA USA; 4 Department of Cardiology, Rigshospitalet, Copenhagen University Hospital, Copenhagen, Denmark; 5 Department of Clinical Medicine, University of Copenhagen, Copenhagen, Denmark

**Keywords:** Cardiac arrest, Coma, Consciousness, Disorders of consciousness, Automated pupillometry, Prognosis, Traumatic brain injury

## Abstract

**Background:**

There is an urgent need for easy-to-perform bedside measures to detect residual consciousness in clinically unresponsive patients with acute brain injury. Interestingly, the sympathetic control of pupil size is thought to be lost in states of unconsciousness. We therefore hypothesized that administration of brimonidine (an alpha-2-adrenergic agonist) eye drops into one eye should produce a pharmacologic Horner’s syndrome if the clinically unresponsive patient is conscious, but not if the patient is unconscious. Here, in a first step to explore this hypothesis, we investigated the potential of brimonidine eye drops to distinguish preserved sympathetic pupillary function in awake volunteers from impairment of sympathetic tone in patients in a coma.

**Methods:**

We enrolled comatose patients admitted for acute brain injury to one of the intensive care units (ICU) of a tertiary referral center, in whom EEG and/or neuroimaging for all practical purposes had ruled out residual consciousness. Exclusion criteria were deep sedation, medications with known drug interactions with brimonidine, and a history of eye disease. Age- and sex-matched healthy and awake volunteers served as controls. We measured pupils of both eyes, under scotopic conditions, at baseline and five times 5–120 min after administering brimonidine into the right eye, using automated pupillometry. Primary outcomes were miosis and anisocoria at the individual and group levels.

**Results:**

We included 15 comatose ICU patients (seven women, mean age 59 ± 13.8 years) and 15 controls (seven women, mean age 55 ± 16.3 years). At 30 min, miosis and anisocoria were seen in all 15 controls (mean difference between the brimonidine-treated pupil and the control pupil: − 1.31 mm, 95% CI [− 1.51; − 1.11], *p* < 0.001), but in none (*p* < 0.001) of the 15 ICU patients (mean difference: 0.09 mm, 95% CI [− 0.12;0.30], *p* > 0.99). This effect was unchanged after 120 min and remained robust in sensitivity analyses correcting for baseline pupil size, age, and room illuminance.

**Conclusion:**

In this proof-of-principle study, brimonidine eye drops produced anisocoria in awake volunteers but not in comatose patients with brain injury. This suggests that automated pupillometry after administration of brimonidine can distinguish between the extremes of the spectrum of consciousness (i.e., fully conscious vs. deeply comatose). A larger study testing the “intermediate zone” of disorders of consciousness in the ICU seems warranted.

**Supplementary information:**

The online version contains supplementary material available at 10.1007/s00701-023-05569-8.

## Introduction

In patients with brain injury, levels of consciousness may fluctuate over days, hours, or even minutes, rendering residual consciousness often difficult to rule in or rule out [14]. Patients can also receive a combination of sedatives and neuromuscular blocking agents that make assessment of the conscious state difficult. Even more challenging is that up to 15% of patients may have partially preserved cognitive abilities although they appear unresponsive at the bedside [15], a condition termed cognitive motor dissociation [27], which is frequent also in the intensive care unit (ICU) [3].

Electroencephalography (EEG) and functional magnetic resonance imaging (MRI) may help to detect residual consciousness, including cognitive motor dissociation [15], but these methodologies are time-consuming, technologically complex, and subject to logistical challenges in the ICU [1]. By contrast, automated pupillometry is a quick and objective bedside measure to examine the pupil [5, 11] with the potential to identify residual consciousness [31].

**Fig. 1:**
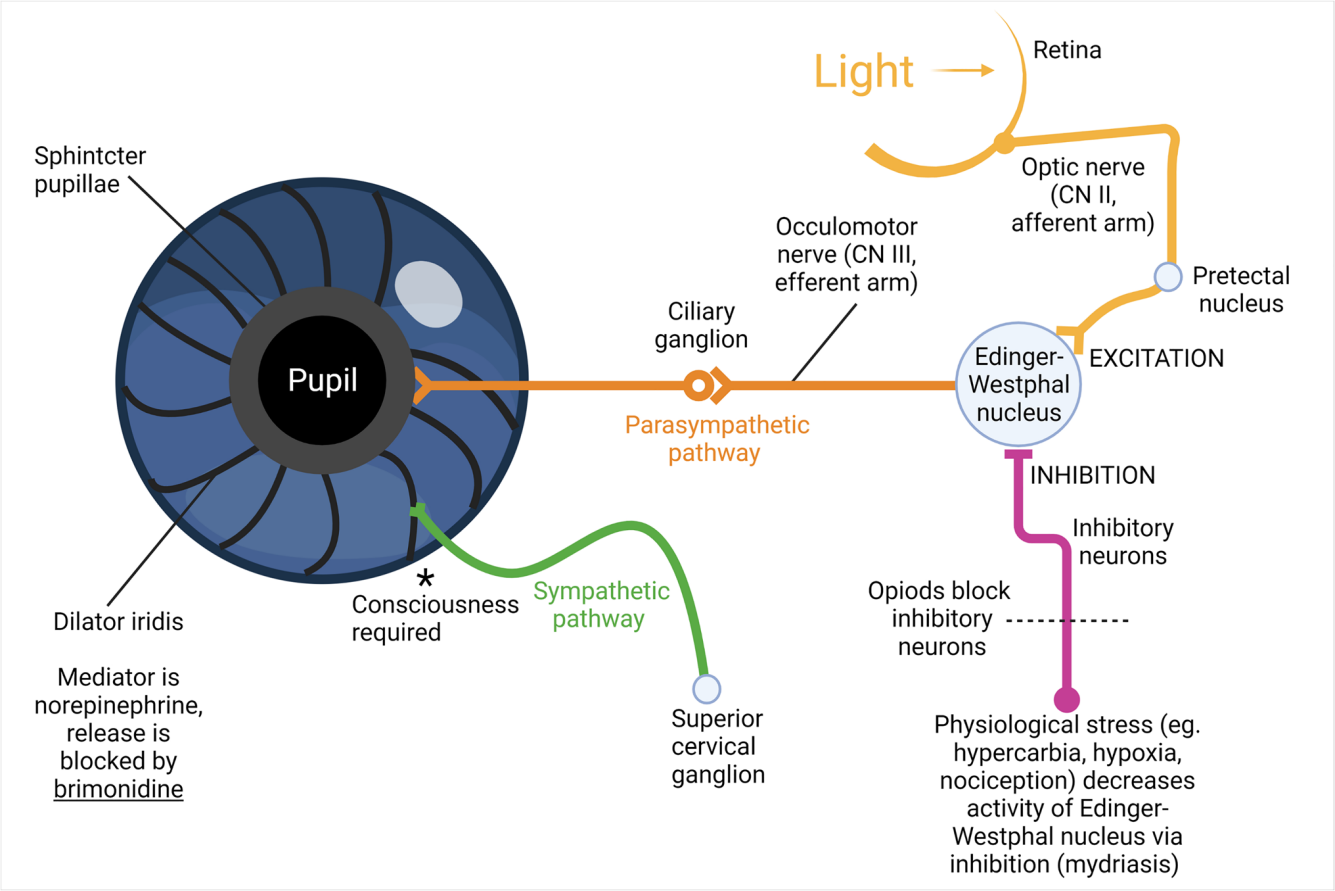
Illustration showing the principal factors that control pupil size. Light excitation of retinal melanopsin ganglion cells activates the neurons of the Edinger-Westphal nucleus. Inhibitory neurons can depress these neurons, and the pupil passively dilates. The preganglionic sympathetic neurons originate in the upper spinal cord, and the postganglionic sympathetic neurons excite the radially oriented muscle to dilate the pupil. However, the sympathetic component is also thought to traverse a pathway above the brainstem, and previous research indicates that consciousness is required for sympathetic tone to be present at the dilator muscle of the iris (asterisk). Our study was designed to confirm or reject experimental studies suggesting that sympathetic tone in the dilator is absent during unconscious states. CN cranial nerve

Pupil size is determined by the two divisions of the autonomic nervous system (Fig. 1). The sympathetic dilator muscle is activated through the intermediolateral cell column in the upper thoracic spinal segments [10]. During conscious states with awareness, pupillary dilation following arousal occurs predominantly through this sympathetic division [33]. The sympathetic component is thought to traverse a pathway above the brainstem [33]. The parasympathetic division originates in the Edinger-Westphal nucleus and innervates the pupillary sphincter. This muscle surrounds the pupillary margin and acts like a purse string to constrict the pupil. There also exists a cortical component of pupillary innervation, with emotional and cognitive load affecting pupil size in awake and healthy volunteers [22].

During anesthesia with loss of awareness, the dilator muscle of the iris does not contribute to resting pupil size and is not activated by nociceptive stimuli. Pupillary dilation in response to nociception continues to occur during anesthesia, but it is brought about solely through inhibition of the Edinger-Westphal nucleus [2, 16–18]. Similarly, in brain dead organ donors with intact spinal sympathetic reflex pathways, an alpha-1-adrenergic inhibitor does not inhibit pupil dilation after a noxious stimulus as it does in healthy volunteers, suggesting a supraspinal component for sympathetic reflex dilation [33]. Taken together, it appears that consciousness is required for sympathetic tone to be present at the dilator muscle of the iris.

**Fig. 2:**
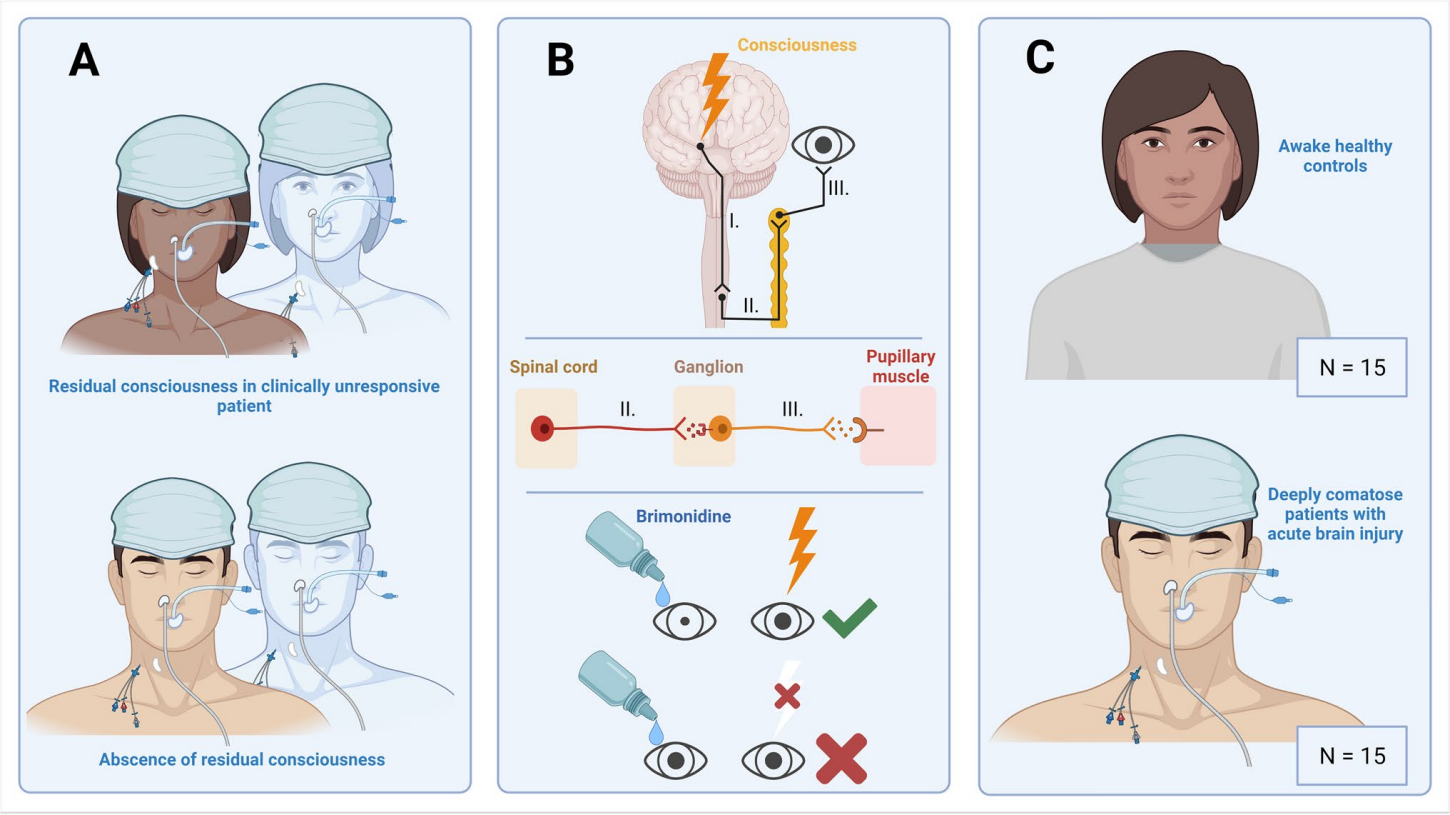
Design and study rationale. **A** Convenient bedside measures to detect residual consciousness in clinically unresponsive patients with acute brain injury are urgently required. **B** Notably, in states of unconsciousness, the sympathetic control of pupil size is thought to be lost. Instillation of brimonidine (an alpha-2-adrenergic agonist) eye drops in one eye should therefore produce miosis and anisocoria if clinically unresponsive patients are conscious (indicated by “lightning”), but not if they are unconscious. **C** In a first step to explore the usefulness of brimonidine eye drops to search for residual consciousness, we investigated the potential of brimonidine to distinguish preserved sympathetic pupillary function in awake volunteers from impairment of sympathetic tone in comatose patients in whom residual consciousness had been ruled out

Evidence from these studies prompted us to ask whether unconsciousness from other causes is associated with loss of sympathetic function in the iris dilator muscle (see Fig. 2 for study rationale and design). Brimonidine is an alpha-2-agonist that reduces sympathetic pupillary tone and decreases intraocular pressure in open-angle glaucoma [9]. If consciousness is necessary for a significant sympathetic pupillary tone to be present [18, 22], then brimonidine eye drops might cause miosis in awake subjects but not in comatose patients (Fig. 2). Developing this idea further, it can be hypothesized that automated pupillometry following brimonidine could aid in detecting the 15% of clinically unresponsive people with brain injury who are in a state of cognitive motor dissociation (and hence have residual consciousness) by revealing preserved pupillary sympathetic tone. Furthermore, it would clarify the presence or absence of consciousness in sedated and paralyzed subjects.

In a first step to explore this hypothesis, we here aimed to falsify it by testing it in subjects at the extremes of the spectrum of consciousness (i.e., fully conscious vs. deeply comatose): If miosis and anisocoria develop after brimonidine treatment both in healthy volunteers and in clinically unresponsive patients with acute brain injury in whom neuroimaging and EEG have ruled out the possibility of residual consciousness, then our hypothesis would be wrong and a larger trial testing the “gray zone” of disorders of consciousness would be unnecessary. By contrast, if results would indicate that automated pupillometry revealing miosis and anisocoria after brimonidine administration can aid in differentiating the extremes of consciousness levels, i.e., awake volunteers vs. deeply comatose patients, then, in a future prospective trial, this technique could be tested as a convenient bedside measure to distinguish levels of consciousness in acute brain injury across the entire spectrum of disorders of consciousness encountered in the ICU.

## Methods

We conducted a prospective study from June 2020 to December 2021 at the ICUs of a tertiary referral center (Rigshospitalet, Copenhagen University Hospital) to investigate the effects of brimonidine eye drops in deeply comatose patients compared to awake and healthy controls.

### Inclusion and exclusion criteria

Comatose patients aged $\ge$ 18 years and admitted to the ICU for acute brain injury following cardiac arrest (n = 8) or acute cerebrovascular disorders (n = 7) were enrolled on a convenience basis when residual consciousness for all practical purposes was ruled out according to clinical exam, neuroimaging (e.g., CT post-cardiac arrest showing global edema with absence of white/gray matter distinction), and/or EEG (e.g., absence of EEG background activity). Exclusion criteria were active eye disease or a history of pupillary injury (e.g., cataract); patients treated with mono-oxidase inhibitors or tricyclic antidepressants due to possible interaction with brimonidine; “high or very high” levels of sedation (see below); and a planned brain death/organ procurement protocol (to avoid interference with brainstem reflex testing).

### Control cohort

Healthy volunteers, matched for age and sex, were recruited from January to March 2022 via local advertisement. Exclusion criteria were active eye disease or a history of pupillary injury and mono-oxidase inhibitors and/or tricyclic antidepressants.

### Baseline assessment

Before pupil measurements, a neurological examination was performed by or under supervision of a board-certified neurologist with > 15 years of experience (DK), which included (but was not limited to) a detailed evaluation of the cranial nerves and brainstem reflexes, Full Outline of UnResponsiveness (FOUR) score [32], and assessment of spontaneous movements (e.g., eye-opening, movement of extremities).

### Pupillary assessment

Room illuminance was measured (in lux) near the eyes using a conventional iPhone and the application “LUX Lightmeter” to ensure that illuminance remained constant throughout all measurements. Ambient light was adjusted/reduced to ensure scotopic conditions (≈25 lx). Pupil size was measured in both the eyes using automated pupillometry (NPi-200 Pupillometer, NeurOptics, USA). Measurements were taken at baseline (T0) before administering a single drop of brimonidine (2 mg/mL) into the right eye to block sympathetic tonus. Measurements were then repeated 5, 10, 20, 30, and 120 min (T5, T10, T20, T30, T120) after intervention, to demonstrate the presence or the absence of anisocoria and miosis. At each time point, the measurement of pupil size was repeated three times for each eye, and mean pupil size was calculated to account for variation.

### Clinical data

Clinical data including cause of admission, MRI, CT, EEG, level of sedation, if applicable, and final diagnosis were collected. Levels of sedation were categorized as “none” (no sedation given during or before the examination); “low to moderate” indicating remifentanil < 15 µg/kg/h, propofol < 2 mg/kg/h, fentanyl < 5 µg/kg/h, sevoflurane < 3%, and midazolam < 0,15 mg/kg/h; and “high to very high” indicating remifentanil > 15 µg/kg/h, propofol > 2 mg/kg/h, fentanyl > 5 µg/kg/h, sevoflurane > 3%, and midazolam > 0,15 mg/kg/h [4].

### Outcomes

Primary outcomes were the following: (1) within-eye difference, measured as mean difference change in pupil size from T0 to T30; (2) between-eye difference, measured as mean difference between the treated (right) and the non-treated (left) pupil in each subject at T30; and (3) between-group difference, measured as a comparison of mean difference change in treated (right) pupil size from T0 to T30 between comatose patients and matched controls. Secondary outcome was correlation of changes in pupil size from T0 to T30 with the FOUR score. T30 was chosen because miosis typically occurs within 30 min after brimonidine administration [12].

### Statistical analysis

Data were assessed by q-q-plot and histogram to check for normal distribution. Baseline and 30-min measurements were not normally distributed, so non-parametric tests were used for further analysis. The primary outcome of within-eye difference was analyzed using Wilcoxon signed-rank test, while the primary outcomes of between-eye and between-group differences were analyzed using Wilcoxon rank-sum test. The secondary outcome (correlation between FOUR score and pupil reactivity) was assessed by Spearman rank correlation. We further performed sensitivity analyses using linear regression models to assess the effect of age, baseline pupil size, and room illuminance on the pupil size at the 30-min measurement, as well as subgroup analysis of the effect of brimonidine after 120 min and another sensitivity analysis of all outcomes, excluding comatose patients with a baseline measurement < 3 mm. Significance was set at *p* < 0.05. Primary outcomes were adjusted for multiple comparisons (*n* = 3) by the Bonferroni correction, and adjusted significance was set at 0.05/3 = 0.016. *p*-values are given after Bonferroni correction. All analyses were performed using R (version 4.2.0., R-packages “summarytools,” “cowplot,” “ggplot2,” “lattice,” “ggpubr,” and “tidyverse”).

### Ethics

The study was approved by the Ethics Committees of the Capital Region of Denmark (file number: H-19044446) and by the Knowledge Center of Data Reviews (file number: P-2021–879). Informed written consent was obtained from the patients’ next-of-kin and a trial guardian (the attending anesthesiologist), as well as from healthy volunteers.

### Data availability

Data is available upon reasonable request.

## Results

### Demographics and baseline neurological assessment

Fifteen comatose patients (mean (SD) age 59 (13.8) years; seven women) and fifteen healthy volunteers (mean (SD) age 55 (16.3) years; seven women) were included (Table 1). All patients had highly pathological brain injury on CT (“highly pathological” being defined as Fisher grade ≥ 3 [subarachnoid hemorrhage], hemorrhage volume ≥ 30 mL [intracerebral hemorrhage], strategic hemorrhage or infarct in brainstem [ischemic stroke or infratentorial hemorrhage], or any visible sign of anoxic brain injury including global or cortical edema [cardiac arrest]). Nine patients had EEGs done, all of which were highly pathological with suppression-burst or continuously suppressed (< 10 µV) background activity. Following a discussion with the patients’ next-of-kin, a decision to withdraw life-sustaining treatment was eventually made in all 15 patients. The mean time from pupillary examination to death was 2.6 days (range: 0–16) with all immediate causes of death being withdrawal of life-sustaining therapy. At baseline (T0), mean (range) FOUR score was 4 (0–9) and mean (range) GCS 3.6 (3–7). During the examination, one patient received high levels of sedation (a protocol violation), six patients received low to moderate levels of sedation, while the remaining eight patients did not receive any sedation. Five patients required inotropic support with norepinephrine before or during examination (Table 2).

**Table 1:** Demographics

	Awake volunteers	Comatose patients	*p*^*a*^
	*N* = *15*	*N* = *15*	
*Sex*			
Female	7 (46.7%)	7 (46.7%)	> 0.99
Male	8 (53.3%)	8 (53.3%)	
*Age mean (IQR)*	54.7 (49–64)	59.2 (50–71.5)	0.429
*Length of ICU stay, days, median (IQR)*	NA	6 (2–8)	NA
*GCS, median (range)*	NA	3 (3–7)	NA
*FOUR, median score (range)*	NA	3 (0–9)	NA
*Time from assessment to death, days, median (IQR)*	NA	1 (0–2.6)	NA
*Levels of sedation*^*b*^	NA		NA
- *None*		9	
- *Low/moderate*		5	
- *High*		1	

Abbreviations: *IQR* interquartile range, *GCS* Glasgow Coma Scale, *FOUR* Full Outline of UnResponsiveness

^a^*t*-test was used for comparisons of means, and Pearson’s *X*^2^ test was used for comparisons of categorical values

^b^ “None” (no sedation given during or before the examination); “low to moderate” indicating remifentanil < 15 µg/kg/h, propofol < 2 mg/kg/h, fentanyl < 5 µg/kg/h, sevoflurane < 3%, and midazolam < 0.15 mg/kg/h; “high to very high” indicating remifentanil > 15 µg/kg/h, propofol > 2 mg/kg/h, fentanyl > 5 µg/kg/h, sevoflurane > 3%, and midazolam > 0.15 mg/kg/h[4]

**Table 2:** Comatose patients, individual data

Study id	Age	Sex	GCS	FOUR*	NPi^a^	Admission cause	Time from study inclusion to death (days)	CT description	Inotropic level µg/kg/h (noradrenaline)	Sedation
A1	72	Male	7	9 (3, 1, 4, 1)	3.7	Anoxic brain injury	3	Cortical and subcortical loss of GM and WM differentiation	8	-
A2	53	Male	3	3 (0, 0, 2, 1)	0	OHCA	0	Loss of GM and WM differentiation and supra- and infratentorial cerebral edema	6	-
A3	65	Female	3	0	0	ICH	0	ICH, MLS, IVH, hydrocephalus, and subfalcine herniation	3	-
A4	77	Male	3	7 (0, 0, 4, 3)	2.4	SAH	0	Hydrocephalus	-	-
A5	74	Female	3	5 (0, 0, 4, 1)	0	SAH	16	SAH, IVH, and bilateral thalamus infarction	-	-
A6	71	Male	5	7 (0, 2, 4, 1)	4.7	IHCA	0	Loss of GM and WM differentiation	-	-
A7	63	Male	5	7 (0, 2, 4, 1)	4.6	OHCA	6	Loss of GM and WM differentiation, cerebral edema, and bilateral infarcts	-	-
A8	73	Female	3	3 (0, 0, 2, 1)	0	ICH	1	Cerebral edema, ICH in basal ganglia, IVH, and hydrocephalus	-	Remifentanil 400 µg/h
A9	45	Female	3	0	0	OHCA	1	Loss of GM and WM differentiation	4	-
A10	23	Male	4	4 (0, 1, 2, 1)	0	OHCA	2	Supratentorial hypoxic ischemic changes	-	Remifentanil 400 µg/h
A11	67	Male	3	7 (0, 0, 4, 3)	3.8	OHCA	2	Loss of GM and WM differentiation, and cerebral edema	-	Midazolam 10 mg × 1 30 min. before examination
A12	69	Female	3	2 (0, 0, 2, 0)	0	Cerebral infarct	1	Loss of GM and WM differentiation and cerebral edema	-	Remifentanil 1000 µg/h
A13	58	Female	3	2 (0, 0, 2, 0)	3.6	ICH	0	Pontine and mesencephalic bleeding, which compromises outflow from 4th ventricle	-	Remifentanil 750 µg/h
A14	47	Female	3	0	0	SAH with OHCA	0	MLS, bioccipital hypodensities, and cerebral edema	-	-
A15	31	Male	3	3 (0, 0, 2, 1)	4.2	Anoxic brain injury	7	Preserved GM and WM differentiation	16	Remifentanil 250 µg/h

Abbreviations: *GCS* Glasgow Coma Scale, *FOUR* Full Outline of UnResponsiveness score, *CT* computer tomography scan, *OHCA* out-of-hospital cardiac arrest, *IHCA* in-hospital cardiac arrest, *ICH* intracerebral hemorrhage, *SAH* subarachnoid hemorrhage, *IVH* intraventricular hemorrhage, *MLS* midline shift, *GM* gray matter, *WM* white matter

^a^NPi = proprietary index for pupillary light response; 0–3, abnormal; > 3–5, normal

^*^Numbers in parenthesis refer to FOUR scores for “eye response,” “motor response,” “brainstem reflexes,” and “respiration,” respectively

### Primary outcomes

#### Between-group difference

**Fig. 3:**
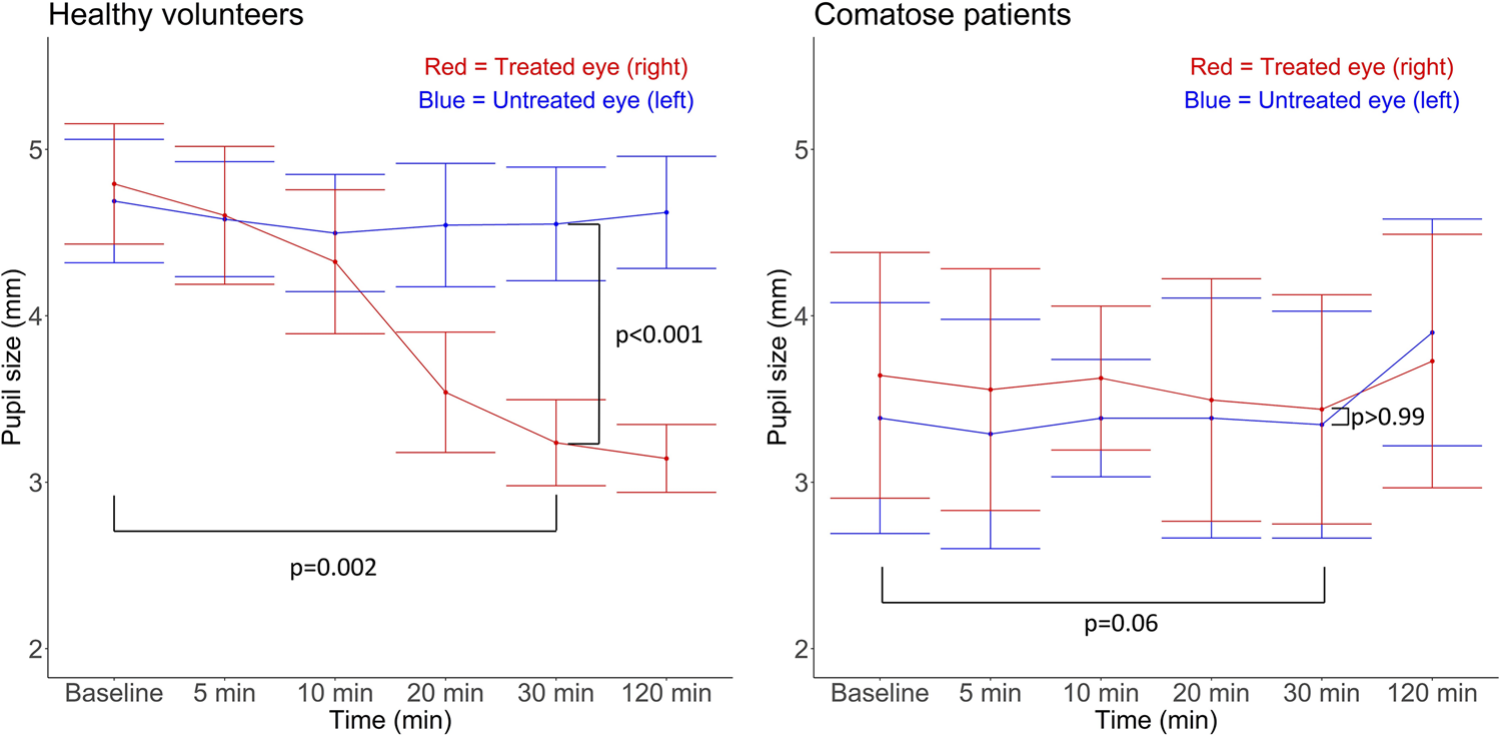
Group data, comatose patients and healthy controls. Mean pupil size over time for healthy volunteers (left) and for comatose patients (right) with 95% CI. Red indicates values for the brimonidine-treated eye (right eye), and blue indicates values for the non-treated eye (left eye). Healthy volunteers showed a significant difference in pupil size from baseline to 30 min after brimonidine administration (*p* = 0.002), as well as a significant difference between the treated (right) and the non-treated (left) eye at the 30-min measurement (*p* < 0.001). Meanwhile, the comatose patients did not show a significant change in pupil size from baseline to 30 min after brimonidine administration (*p* = 0.06) nor between the treated (right) and the non-treated (left) eye at 30 min (*p* > 0.99)

Compared to the comatose group, the control group had a larger decrease in pupil size in the brimonidine-treated eye compared to the comatose group with a mean difference between groups of 1.35 mm (95% CI: [1.30; 1.40], *p* < 0.001) at T30 (Fig. 3). Anisocoria/miosis was observed in all 15 healthy volunteers but in none of the 15 comatose patients (*p* < 0.001).

#### Within-eye difference

**Fig. 4:**
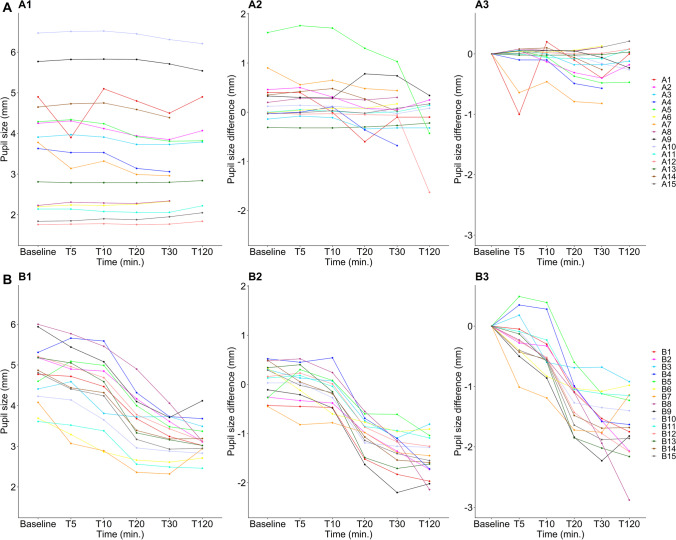
Individual data, comatose patients (**A**). Graphs with data from comatose patients (see legend to the right for patient specification). Pupil size over time (A1), for all 15 comatose patients. Five patients have missing values for the 120-min measurement. Difference in pupil size between treated eye (right eye) and non-treated eye (left eye) (A2), and absolute difference in pupil size from baseline (A3). Individual data, healthy controls (**B**). Graphs with data from healthy volunteers (see legend to the right for patient specification). Pupil size over time (B1), for all 15 healthy volunteers. Difference in pupil size between treated eye (right eye) and non-treated eye (left eye) (B2) and absolute difference in pupil size from baseline (B3)

In the eye of intervention, a significant decrease in mean pupil size from T0 to T30 was seen in the control group (mean change =  − 1.56 mm, 95% CI: [− 1.77; − 1.34], *p* = 0.002) but not in the comatose group (mean change =  − 0.20 mm, 95% CI: [− 0.35; − 0.06], *p* = 0.06). No significant change in pupil size from T0 to T30 was seen in the control eye in either group (Figs. 3 and 4).

#### Between-eye difference

Between the brimonidine-treated eye and the control eye at T30, a significant difference was seen in the control group (mean difference =  − 1.31 mm, 95% CI: [− 1.51; − 1.11], *p* < 0.001) but not in the comatose group (mean difference = 0.09 mm, 95% CI: [− 0.12; 0.30], *p* > 0.99; Figs. 3 and 4).

### Secondary outcome and sensitivity analyses

**Fig. 5:**
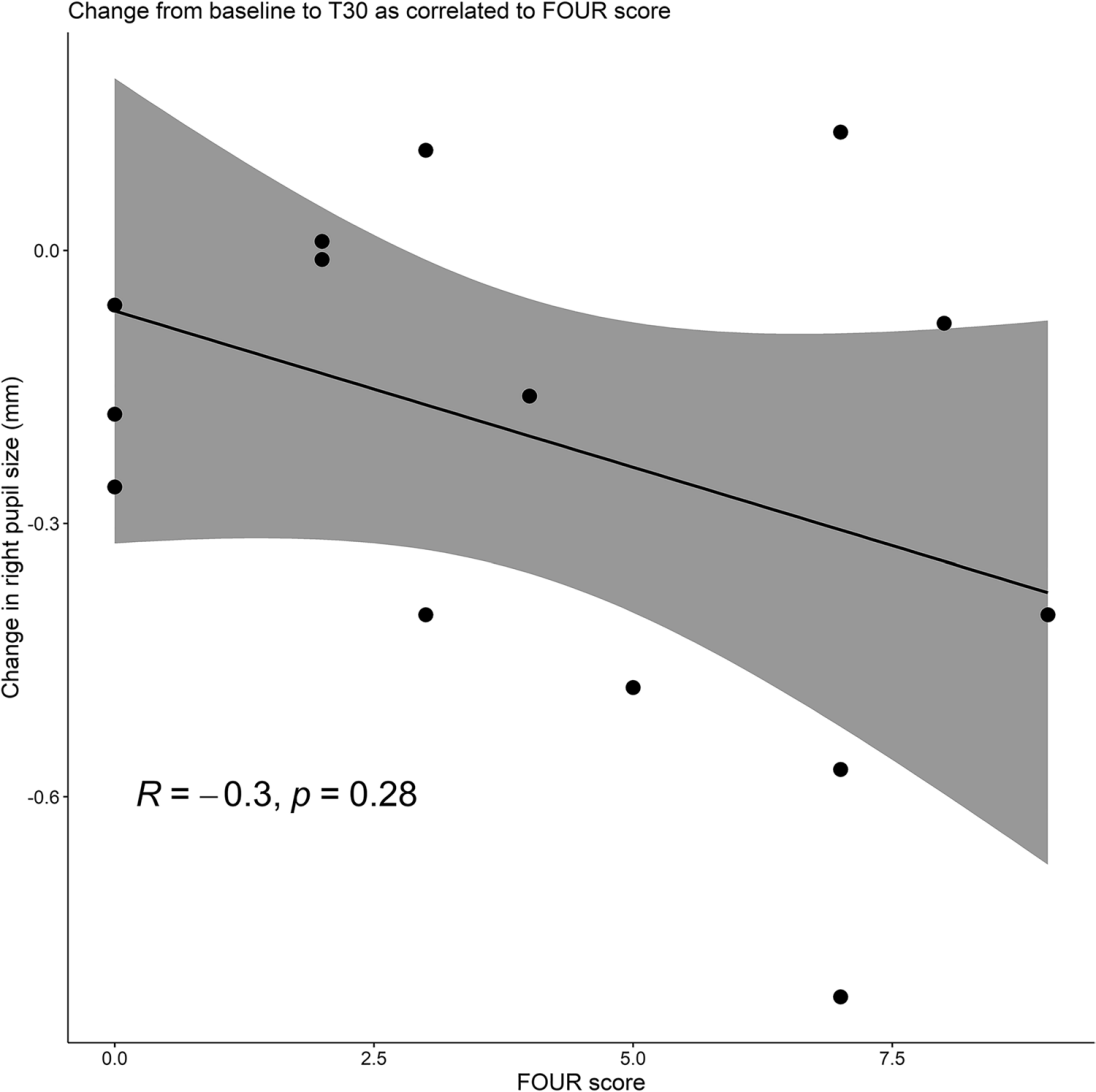
Individual pupillary sizes related to FOUR scores in comatose patients. Change in treated (right) pupil size from T0 to T30 as correlated to FOUR score

In comatose patients, the change of pupil size from T0 to T30 (i.e., the pupillary reactivity to brimonidine) did not correlate with the FOUR scores (*R* =  − 0.3, *p* = 0.28; Fig. 5). There was a significant difference in pupil size change from T0 to T30 and T120 in the control group compared to the comatose group (Table 3). This difference was not affected by age or room illuminance. An association was observed between pupil size at T0 and the change in pupil size at T30 and T120, with larger pupil size at T0 resulting in a greater difference at T30 and T120 (Table 3).

**Table 3:** Linear regression model of change in pupil size and age, lux^a^, and baseline pupil size on the brimonidine treated eye

	Intercept (30 min)		Intercept (120 min)	
	Mean	*P*-value	Mean	*p*-value
*Age*	− 0.01	0.06	− 0.008	0.21
*Lux*	0.003	0.44	0.004	0.51
*Baseline*	− 0.17	0.008	− 0.233	0.006

This sensitivity analysis shows that the difference in pupil size change from T0 to T30 and T120 in the volunteers compared to the comatose patients was not affected by age or room illuminance. A larger pupil size at T0 resulted in a larger difference at T30 and T120

^a^Room illuminance

All healthy volunteers had a baseline pupil size > 3 mm, while only nine of the comatose patients had a baseline pupil size > 3 mm. To account for this difference, we did a sensitivity analysis of all outcomes, excluding comatose patients with a baseline measurement < 3 mm (*n* = 6). This analysis showed that the control group still had a larger decrease in pupil size compared to the comatose group with a mean difference between groups of 1.16 mm, 95% CI: [1.08; 1.23], *p* < 0.001. The comatose group showed a significant change in pupil size from T0 to T30 in the treated eye, yet the effect of brimonidine was still small with a mean change of − 0.37 mm, 95% CI: [− 0.52; − 0.21], *p* = 0.012. Importantly, the difference between the treated and the non-treated eye remained non-significant at T30 with a mean difference of 0.13 mm, 95% CI: [− 0.21; 0.47], *p* = 0.93. Thus, the significant difference in the treated eye also occurred in the non-treated eye (Supplementary Table S1).

## Discussion

In this study, we looked for miosis and anisocoria after one drop of brimonidine into the right eye of subjects at the extremes of consciousness levels, that is, fully conscious vs. deeply comatose. We found a significant and persisting decrease in pupil size in treated compared to non-treated pupils in healthy volunteers but not in deeply comatose patients with acute brain injury. The presence of miosis and anisocoria indicates normal sympathetic pupillary function in healthy volunteers, whereas their absence implies a lack of sympathetic tone in comatose patients with acute brain injury. We were hence unable to falsify our hypothesis that automated pupillometry might be a tool to identify residual consciousness in unresponsive patients with brain injury, including those with cognitive motor dissociation. We conclude that a trial across the full range of disorders of consciousness encountered in the ICU could be meaningful.

Intravenous administration of alpha-1-adrenergic agonists such as norepinephrine and epinephrine do not change pupil size in humans [16]. In contrast, release of norepinephrine from the postganglionic sympathetic nerves results in contraction of the dilator muscle and dilation of the pupil. Brimonidine blocks sympathetic tone at the level of the iris by activating the alpha-2-adrenergic receptors on the sympathetic terminals [9]. These receptors exert negative feedback resulting in reduced sympathetic tone, with inhibition of the release of norepinephrine, iris dilator muscle relaxation, and pupillary constriction. It is important to understand that if there is sympathetic tone in the dilator muscle of the iris, then the pupil will constrict after the application of brimonidine. The absence of a light reflex would not alter the constriction. Loewenfeld’s classic book on the physiology and clinical applications of the pupil [18] displays a list of over 100 references, showing that the sympathetic dilator muscle can alter pupil size after total parasympathetic paralysis (with loss of the light reflex). For instance, cervical sympathectomy contracts the pupil after parasympathetic denervation. By contrast, pupillary dilation occurs after stimulation of the cervical sympathetic when the third nerve innervation of the sphincter is blocked. It is a common clinical observation that the induction of anesthesia constricts the tropicamide-dilated pupil [16]. This occurs, as further outlined below, because the sympathetic tone in the dilator is lost with the onset of unconsciousness.

Devastating brain injury can lead to loss/diminution of sympathetic tone, but can we ascribe our results to patients’ conscious state and not to the loss of other brain functions, notably in patients with direct brainstem involvement? We think we can. The explanation is that for over 60 years, neuroscientists have demonstrated that the dilator muscle of the iris does not contribute to pupil size or to reflex dilation during unconscious states [2, 16–19, 33]. The other portions of the sympathetic nervous system that control vasoconstriction and heart rate remain largely intact (even though there are data regarding heart rate variability that show that some metrics scale with consciousness state, e.g., [23]). Several articles have confirmed that blood pressure and heart rate changes often occur following the skin incision during organ procurement (see [18] and references therein). This can be a dramatic change and it happens without any sympathetically mediated dilation of the pupil [18]. Presumably these hemodynamic changes occur because of direct connection of nociceptive afferents onto the spinal cord sympathetic neurons in the upper spinal cord. This happens in brain dead subjects without brainstem function and would certainly be obvious in (comatose, vegetative state, or minimally conscious) patients with extensive cortical damage but preserved brainstem function. We are not aware of studies that have consistently demonstrated loss of sympathetic tone in the dilator muscle of the iris after major stroke or cardiac arrest without loss of consciousness. Extensive brainstem damage would very likely result in loss of consciousness. We therefore cannot conclude that our results are simply the result of devastating brain injury, but they are likely a function of the conscious state.

It therefore appears that given automated pupillometry and brimonidine eye drops, this relatively simple neurological framework can potentially be leveraged to identify residual consciousness in clinically unresponsive patients with brain injury in the ICU. This would be a major achievement for several reasons: First, recovery of consciousness is the single most important determinant of clinical outcome after brain injury and coma; yet neuroprognostication is notoriously difficult [6, 8, 20, 26]. EEG and MRI can assist in determining consciousness levels but pose logistical challenges in the ICU and the need for technological expertise [1]. Second, 15% of patients who appear clinically unresponsive are indeed awake and alert and can follow commands in sophisticated mental tasks during EEG- and functional MRI-based paradigms, but these paradigms are not available in clinical routine yet [15]. Comparable states of cognitive motor dissociation are known to occur with similar frequency in the ICU [3], where EEG- and fMRI-based consciousness paradigms are even more difficult to conduct [7, 14]. Third, as seven of ten deaths in the ICU occur after a decision is made to withdraw life-sustaining therapy [30], missing residual consciousness has major implications for medical decision-making in the ICU, including prognostication, rehabilitation, resource allocation, end-of-life decisions, and caregiver well-being [34]. Given that every year, on a population level 2 out of 1000 people enter a coma [13], there is an urgent need for better prognostic tools in the ICU. It follows that an easy-to-perform, unexpensive point-of-care bedside approach like automated pupillometry combined with brimonidine eye drops would have many benefits.

### Strengths and limitations

This study has limitations. It appears that the pupil did constrict in some of the comatose patients after brimonidine (Fig. 4A), but this effect if present was minor and temporary compared to that in awake volunteers (Fig. 4B). There are several possible explanations. First, tonic sympathetic tone of the dilator muscle of the iris depends on the viability of brainstem structures that activate the preganglionic sympathetics in the upper spinal cord [24, 25]. It is possible that with these cases, there was a small residual activation of the dilator from brainstem centers. For example, the locus coeruleus and other brainstem nuclei have descending projections to the intermediolateral cell column [24, 25]. These brainstem pathways likely remained partially intact in at least some of the cases we studied. We cannot rule out that a change in the intracranial pathology occurred over the 2-h study period, but we deem this unlikely. Second, it might be that the alpha-1 receptor at the dilator muscle had developed a small degree of denervation hypersensitivity during the days after the insult and before the study was conducted [28]. Even a small release of norepinephrine from the postganglionic sympathetic nerves might then produce a residual activation of the dilator that was blocked by brimonidine. Third, brimonidine also decreases aqueous humor production and thereby decreases intraocular pressure, which comes about by a direct alpha 2 agonist effect on the blood vessels. This effect is not related to the alpha 2 activity that prevents norepinephrine release from the sympathetic nerves. A decrease in intraocular pressure might therefore result in a very small decrease in pupil diameter, and in fact, the use of apraclonidine (instead of brimonidine) to diagnose Horner’s syndrome may circumvent this physiological effect [29].

The pupillary responses we report were highly variable. However, we purposely studied a diverse population of unconscious subjects to avoid limiting our study to a specific brain injury. While this did produce variable pupillary responses, our statistical analysis of this exploratory study confirmed our hypothesis that the unconscious state is associated with lack of tone in the dilator muscle of the iris. A larger cohort is needed to specifically investigate pupillary responses in coma related to strategic brainstem lesions, bi-hemispheric damage, and global metabolic/anoxic compromise, respectively.

The comatose patients had various types of brain injury because they were selected to reflect a real-life ICU setting of acute brain injuries. Since the aim was to test a consciousness biomarker that would be meaningful and reliable across the entire range of brain injuries encountered in the ICU, the comatose group was, as stated above, heterogeneous on purpose. In other words, a consciousness marker that only would work in, say, patients with a focal frontal lesion owing to traumatic brain injury, but not in patients with parietal lesions or those after cardiac arrest, would be of little practical value. We acknowledge that the cohort was small, and a larger prospective validation trial is needed to confirm that the results are generalizable, but—as stated—in this pilot study, we focused on the extremes of the consciousness levels to test if our primary hypothesis could be falsified. This approach required a comparably smaller sample size.

The challenging setting in the ICU, with critically ill patients who are subject to sudden deterioration, also resulted in missing values for the 120-min measurement for five of the 15 patients, but we do not think that the lack of this data compromises the overall findings of the study. Also, despite our exclusion criteria, one patient received high levels of sedation at the time of pupillary exam, which was a protocol violation, but we decided to keep this patient in the cohort; nevertheless, given the naturalistic ICU setting. Critically ill patients with acute brain injuries typically require use of sedatives, inotropes, and opioids, and increased intracranial pressure may affect third cranial nerve functions. Notably, coma patients with small pupils were mostly those that received remifentanil, and opioid constricted pupils have a tightly constricted sphincter muscle. However, sensitivity analysis for baseline pupil size showed that the brimonidine effect was minimal in comatose patients with pupil sizes of 3 mm or more. Still, all these factors must be considered when pupillometry is used for prognostication, as they can have a confounding effect [21], but a method to identify residual consciousness in the ICU that would be prone to major artifacts would have very limited clinical utility.

We assessed comatose patients with the FOUR score, which works well with intubated patients [32]. Scores ranged from 0 to 9 points, indicating a difference in wakefulness. However, FOUR scores were not significantly correlated to the difference in pupil size between baseline and 30 min after intervention, possibly owing to the small sample size and lack of statistical power.

Finally, the presence of sympathetic tone may be necessary for consciousness but may not be sufficient. However, all sympathetic tone is not abolished during unconsciousness. We here are referring only to sympathetic tone in the dilator muscle of the iris. Patients with a preexisting Horner’s syndrome could be conscious but would not constrict following topical administration of brimonidine. Also, patients with tonic pupils might not exhibit any change in size after brimonidine. Because no tests are perfect 100% of the time, a more complete study is required to evaluate the specificity and sensitivity of the brimonidine test for the unconscious state.

The major strengths of this study are the age- and sex-matched groups, control of room illuminance, which ensured scotopic conditions, and repeated measurements over 120 min with automated pupillometry. These factors are important as age can affect pupil rigidity, and brimonidine has a larger effect under scotopic conditions, so ensuring low room illuminance was crucial [12]. With repeated measurements, the probability of overlooking an even short-lasting miotic effect was diminished. Also, for the purpose of this proof-of-concept study, even though evoked potentials and advanced fMRI/EEG-based consciousness paradigms were not performed, we ensured that clinical exam, EEG, and neuroimaging had rendered the presence of residual consciousness close to impossible, despite no or limited sedation. Furthermore, linear regression models revealed that our results remained robust when corrected for baseline pupil size, age, and room illuminance.

## Conclusions

Our results indicate that automated pupillometry revealing miosis and anisocoria after brimonidine administration can aid in differentiating the extremes of consciousness levels, i.e., awake volunteers vs. deeply comatose patients, within 30 min. In a future prospective (multicenter) trial, this technique could be tested to distinguish levels of consciousness in acute brain injury across the entire spectrum of disorders of consciousness encountered in the ICU. Such a trial should be done with mental task-based fMRI and EEG paradigms as the gold standard to identify ICU patients in cognitive motor dissociation.

## Supplementary information

Below is the link to the electronic supplementary material.

Supplementary file1 (DOCX 15 KB)
