# Supplementary material for: Brimonidine eye drops reveal diminished sympathetic pupillary tone in comatose patients with brain injury
Source: Acta Neurochir (Wien). 2023 Apr 4;165(6):1483–94. doi: 10.1007/s00701-023-05569-8 (PMC10227128; doi:10.1007/s00701-023-05569-8)
Supplement: Supplementary file 1 — Supplementary file1 (DOCX 15 KB) [file 701_2023_5569_MOESM1_ESM.docx]

| **Table S1. Sensitivity analysis excluding comatose patients with baseline pupil size <3 mm.** | | | |
| --- | --- | --- | --- |
|  | **Within-eye from T0 to T30** | **Between-eye** | **Between-groups at T30** |
| **Healthy volunteers** | -1.56 mm, 95% CI: [-1.77;-1.34], p=0.002 | - 1.31 mm, 95% CI: [-1.51;-1.11], p<0.001 | 1.35 mm, 95% CI: [1.30;1.40], p<0.001) |
| **Comatose patients** | -0.20 mm, 95% CI: [-0.35;-0.06], p=0.06 | 0.09 mm, 95% CI: [-0.12;0.30], p>0.99 |  |
| **Coma patients excluding T0<3mm (n=6)** | -0.37 mm, 95% CI: [-0.52;-0.21], p=0.012 | 0.13 mm, 95% CI: [-0.21;0.47], p=0.93 | 1.16 mm, 95% CI: [1.08;1.23], p<0.001 |
